# Supplementary material for: Thermosensory behaviors of the free-living life stages of Strongyloides species support parasitism in tropical environments
Source: bioRxiv. 2024 Sep 15:2024.09.12.612595. Preprint. [Version 2] doi: 10.1101/2024.09.12.612595 (PMC11419086; doi:10.1101/2024.09.12.612595)

bioRxiv preprint doi: https://doi.org/10.1101/2024.09.12.612595; this version posted September 15, 2024. The copyright holder for this preprint (which was not certified by peer review) is the author/funder, who has granted bioRxiv a license to display the preprint in perpetuity. It is made available under aCC-BY 4.0 International license.

Supplemental Information

Figure S1: Life cycles of *S. stercoralis* and *S. ratti*

A) The life cycle of *S. stercoralis* in a human host [8]. 1) Infection starts when soil-dwelling infective larvae (iL3s) locate a host and skin penetrate. 2) Inside the host, iL3s migrate to the small intestine and develop into reproductively active parasitic females. The eggs and larvae of parasitic females are voided from the host in feces, where they can either develop directly into iL3s (3a) or into non-parasitic “free-living” adults that engage in sexual reproduction (3b). The offspring of the free-living adults all develop into iL3s. Autoinfection can occur when eggs hatch within the host and develop into iL3s.

bioRxiv preprint doi: https://doi.org/10.1101/2024.09.12.612595; this version posted September 15, 2024. The copyright holder for this preprint (which was not certified by peer review) is the author/funder, who has granted bioRxiv a license to display the preprint in perpetuity. It is made available under aCC-BY 4.0 International license.

677 B) The life cycle of *S. ratti* in a rat host [19]. The stages of infection are similar to *S. stercoralis*,
678 with the following differences: a) the progeny of parasitic females leave the host exclusively as
679 unhatched eggs; b) the lack of precocious hatching precludes autoinfection of original hosts.
680
681 **Figure S2: *Strongyloides* free-living males display thermal preferences similar to free-**
682 **living females**
683 A) Individual tracks of *S. stercoralis* free-living males responding to a 20-25°C temperature
684 gradient (T_{start} = 23°C). T_C = 20°C. Each colored line is the track of an individual *S. stercoralis*
685 male's path throughout the 45-minute assay. Black crosses represent the starting positions of
686 each worm. The grey line represents T_{start} = 23°C.
687 B) Individual tracks of *S. ratti* free-living males responding to a temperature gradient from 20°C
688 to 25°C when placed at 23°C. Each colored line is the track of an individual *S. stercoralis* male's
689 path throughout the 45-minute assay. Black crosses represent the starting positions of each
690 worm. The grey bar represents a 1°C-wide starting zone of the assay and is centered on 23°C.
691 C) Quantification of the change in temperature (left) and mean speed (right) for free-living males
692 (FLMs) vs. free-living females (FLFs) of *S. stercoralis* (purple) or *S. ratti* (teal). Icons indicate
693 responses of individual worms, boxes show medians and interquartile ranges, and whiskers
694 show min and max values. *S. stercoralis* FLMs and FLFs migrated similarly in the temperature
695 gradient; *S. ratti* FLMs showed reduced positive thermotaxis relative to *S. ratti* FLFs. The mean
696 speed of worms in the gradient was not significant between the sexes. n = 76 worms for
697 *S. stercoralis* FLFs (7 assays over 5 days), n = 49 worms for *S. stercoralis* FLMs (5 assays over
698 3 days), n = 59 for *S. ratti* FLFs (6 assays over 4 days), and n = 49 for *S. ratti* FLMs (5 assays
699 over 4 days). Icons indicate responses of individual worms, boxes show medians and

bioRxiv preprint doi: https://doi.org/10.1101/2024.09.12.612595; this version posted September 15, 2024. The copyright holder for this preprint (which was not certified by peer review) is the author/funder, who has granted bioRxiv a license to display the preprint in perpetuity. It is made available under aCC-BY 4.0 International license.

interquartile ranges, and whiskers show min and max values. ns = not significant, ***p<0.001, two-way ANOVA with Šídák's multiple comparisons test.

D) Categorical distribution of thermotaxis behaviors in a ~21-35°C gradient across species. Individuals were considered to have engaged in positive or negative thermotaxis if their position at the end of the assay was outside of a 1 cm neutral exclusion zone centered on the starting position of each individual worm. Individuals that finished the assay within this zone were considered non-responding. ns = not significant, *p<0.05, Fisher's exact test with Bonferroni-Dunn correction for multiple comparisons.

Figure S3: *S. stercoralis* FLFs fail to display noxious heat escape behaviors at host body temperatures.

A) Individual tracks of *S. stercoralis* free-living females responding to a ~32-40°C temperature gradient (T_{start} = 38°C). T_C = 23°C. Each colored line is the trajectory of an individual worm during the 45-minute assay. Black crosses represent the starting positions of each worm. The grey line represents T_{start} = 38°C.

B) Quantification of the change in temperature for *S. stercoralis* free-living females. Values are final temperature – starting temperature for each worm. Icons indicate responses of individual worms; boxes show medians and interquartile ranges; whiskers show min and max values. ns = not significantly different from a hypothetical value of 0, Wilcoxon signed-rank test.

C) Categorical distribution of *S. stercoralis* thermotaxis behavior in a ~32-40°C gradient. Individuals were considered to have engaged in positive or negative thermotaxis if their position at the end of the assay was outside of a 1 cm neutral exclusion zone centered on the starting position of each individual worm. Individuals that finished the assay within this zone were considered non-responding.

bioRxiv preprint doi: https://doi.org/10.1101/2024.09.12.612595; this version posted September 15, 2024. The copyright holder for this preprint (which was not certified by peer review) is the author/funder, who has granted bioRxiv a license to display the preprint in perpetuity. It is made available under aCC-BY 4.0 International license.

Figure S4: Differences in thermotaxis navigation between *Strongyloides* iL3s and FLFs

A) Tracks of *S. stercoralis* and *S. ratti* iL3s engaging in positive thermotaxis in a ~21-34°C temperature gradient (T_{start} = 30°C, T_C = 23°C). Each colored line is a track of an individual worm's path throughout the 15-minute assay. Black crosses represent the starting positions of each worm. The grey line represents T_{start} = 30°C.

B) Quantification of the change in temperature (left), mean speed (center), and distance ratio (right) for *S. stercoralis* iL3s and FLFs (purple), and *S. ratti* iL3s and FLFs (teal). Distance ratio was calculated by dividing the total path length by the distance from starting position to ending position of the assay. A larger distance ratio represents a more circuitous path; a distance ratio of 1 is a completely straight path. Icons indicate responses of individual worms, boxes show medians and interquartile ranges, and whiskers show min and max values. n=30-60 worms from 3-6 assays. ns = not significant, ***p<0.001, ****p<0.0001, Kruskal-Wallis test with Dunn's multiple comparisons test.

Figure S5: Conditions that elicit negative thermotaxis in *Strongyloides* iL3s do not elicit negative thermotaxis in *Strongyloides* FLFs.

A-D) Tracks of *S. stercoralis* iL3s (A), *S. stercoralis* FLFs (B), *S. ratti* iL3s (C), and *S. ratti* FLFs (D) in a ~13-23°C gradient (T_{start} = 20°C, T_C = 23°C). Each colored line is an individual worm's path throughout the 15-minute assay. Black crosses represent the starting positions of each worm. The grey line represents T_{start} = 20°C. Assay duration: 45 minutes (adults) or 15 minutes (iL3s).

E) Quantification of the change in temperature experienced by worms in the ~13-23°C gradient. Icons indicate responses of individual worms, boxes show medians and interquartile ranges,

bioRxiv preprint doi: https://doi.org/10.1101/2024.09.12.612595; this version posted September 15, 2024. The copyright holder for this preprint (which was not certified by peer review) is the author/funder, who has granted bioRxiv a license to display the preprint in perpetuity. It is made available under aCC-BY 4.0 International license.

and whiskers show min and max values. n = 30-50 worms from 3-5 assays. ***p<0.001, ****p<0.0001, two-way ANOVA with Tukey's multiple comparisons test.

F) Categorical distribution of thermotaxis behaviors in a ~13°C-23°C gradient across species. Individuals were considered to have engaged in positive or negative thermotaxis if their position at the end of the assay was above or below 0.5°C of their starting position respectively. Individuals that finished the assay within 0.5°C above or below T_{start} were considered non-responding. ***p<0.001, Fisher's exact test with Bonferroni-Dunn correction for multiple tests.

Figure S6: Expanded quantifications of the impact of an attractive odorant on the thermotaxis behaviors of *S. stercoralis* FLFs.

A) Tracks of worms in a ~21-25°C pure thermotaxis gradient or a thermal gradient with an attractive odorant (3m1b, pure) placed near (at 22.5°C) or far (at 22°C) from T_{start} (23°C). Assay duration: 45 minutes. Colored tracks represent the path of individual worms. Black crosses represent the starting location of individual worms. The grey line represents T_{start} = 23°C.

B) Representative tracks of individual *S. stercoralis* FLFs in each experimental condition from panel A. Tracks are color-coded by time. Vertical red hash lines represent the position of the odorant. Black crosses show the starting location of individual worms.

C) Quantification of the minimum temperature experienced by *S. stercoralis* FLFs in a ~21-25°C gradient with and without 3m1b. The minimum temperature experienced in the presence of an odorant was significantly lower than in the temperature-only condition. Icons indicate responses of individual worms, boxes show medians and interquartile ranges, and whiskers show min and max values. ns = not significant, ***p<0.001, ****p<0.0001, Kruskal-Wallis test with Dunn's multiple comparisons test.

bioRxiv preprint doi: https://doi.org/10.1101/2024.09.12.612595; this version posted September 15, 2024. The copyright holder for this preprint (which was not certified by peer review) is the author/funder, who has granted bioRxiv a license to display the preprint in perpetuity. It is made available under aCC-BY 4.0 International license.

D) Quantification of the maximum temperature experienced by *S. stercoralis* FLFs in a ~21-25°C gradient with and without 3m1b. The maximum temperature experienced in the presence of an odorant was significantly lower than in the temperature-only condition. n = 76 worms for temperature only (7 assays over 5 days), n = 65 worms for odorant near (6 assays over 3 days), and n = 66 worms for odorant far (6 assays over 3 days). *p<0.05, ****p<0.0001, Kruskal-Wallis test with Dunn's multiple comparisons test.

E) Quantification of the starting temperature experienced by *S. stercoralis* FLFs *stercoralis* a ~21-25°C gradient with and without 3m1b. The starting temperatures of worms were not significantly different between the experimental conditions. ns = not significant, Kruskal-Wallis test with Dunn's multiple comparisons test.

Figure S7: Kaplan-Meier survival curves for individual experiments.

Probability of survival over time for *C. elegans* adult hermaphrodites, *S. stercoralis* FLFs, and *S. ratti* FLFs. Plots show survival curves for independent experiments. n = number of events (number of censored worms). When the number of censored worms is marked with an asterisk (*), the date of censoring is unknown, and the censored worms are excluded from plots and statistical analyses.

Figure S8: Impact of environmental temperature on daily brood sizes across species.

A-C) Brood size (eggs + larvae) recorded each day for *C. elegans* adults (A), *S. stercoralis* FLFs (B), and *S. ratti* FLFs (C) as a function of incubation temperature. Grey circles = 23°C; yellow diamonds = 30°C; red triangles = 37°C. Icons indicate brood sizes of individual worms, boxes show medians and interquartile ranges, and whiskers show min and max values.

bioRxiv preprint doi: https://doi.org/10.1101/2024.09.12.612595; this version posted September 15, 2024. The copyright holder for this preprint (which was not certified by peer review) is the author/funder, who has granted bioRxiv a license to display the preprint in perpetuity. It is made available under aCC-BY 4.0 International license.

D) Day one brood size across species. *S. stercoralis* FLFs showed a significant increase in brood size on day 1 of the brood size assay at 30°C and 37°C. *S. ratti* FLFs showed a significant increase in brood size on day 1 of the brood size assay at only 30°C. In contrast, *C. elegans* adults showed a significant decrease in brood size on day 1. n = 31-54 adult worms. ns = not significant, ****$p<0.0001$, two-way ANOVA with Tukey's multiple comparisons test.

Supplemental File 1: This file includes all data used for statistical analyses and the results of statistical tests, including exact *p* values.

bioRxiv preprint doi: https://doi.org/10.1101/2024.09.12.612595; this version posted September 15, 2024. The copyright holder for this preprint (which was not certified by peer review) is the author/funder, who has granted bioRxiv a license to display the preprint in perpetuity. It is made available under aCC-BY 4.0 International license.

Figure S1

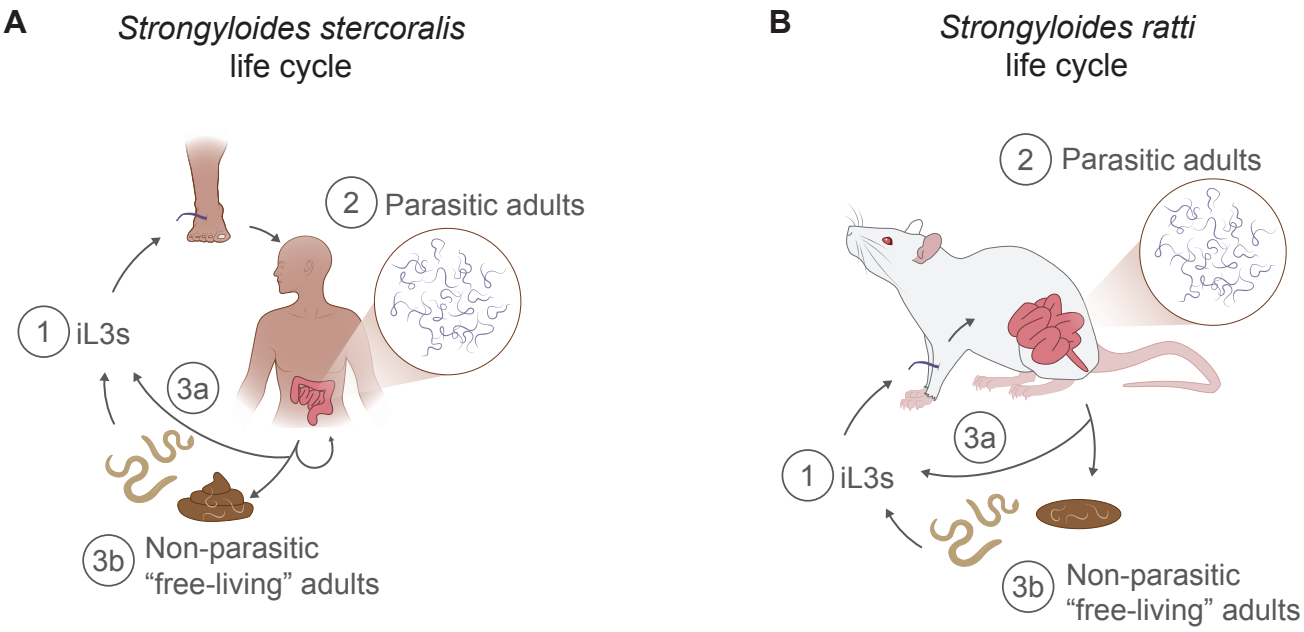

bioRxiv preprint doi: https://doi.org/10.1101/2024.09.12.612595; this version posted September 15, 2024. The copyright holder for this preprint (which was not certified by peer review) is the author/funder, who has granted bioRxiv a license to display the preprint in perpetuity. It is made available under aCC-BY 4.0 International license.

Figure S2

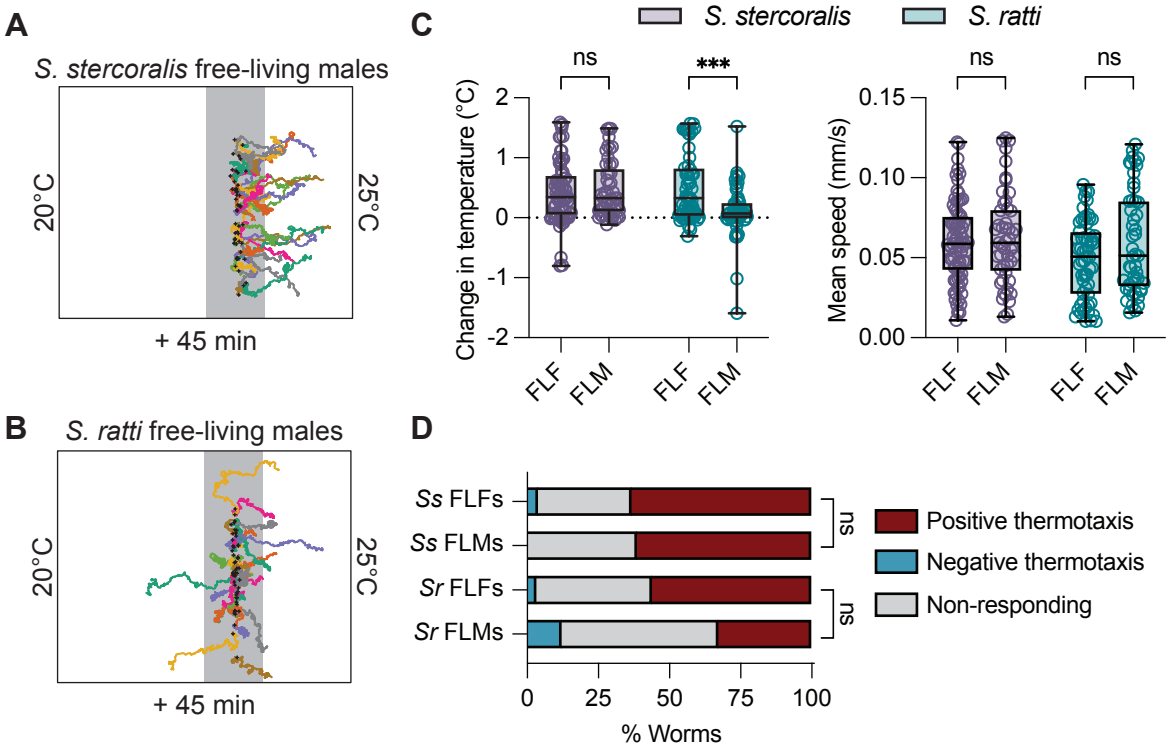

bioRxiv preprint doi: https://doi.org/10.1101/2024.09.12.612595; this version posted September 15, 2024. The copyright holder for this preprint (which was not certified by peer review) is the author/funder, who has granted bioRxiv a license to display the preprint in perpetuity. It is made available under aCC-BY 4.0 International license.

Figure S3

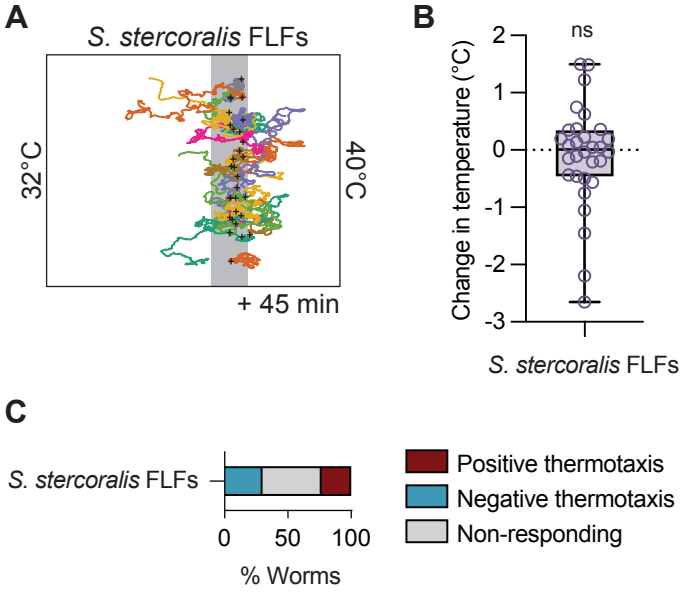

bioRxiv preprint doi: https://doi.org/10.1101/2024.09.12.612595; this version posted September 15, 2024. The copyright holder for this preprint (which was not certified by peer review) is the author/funder, who has granted bioRxiv a license to display the preprint in perpetuity. It is made available under aCC-BY 4.0 International license.

Figure S4

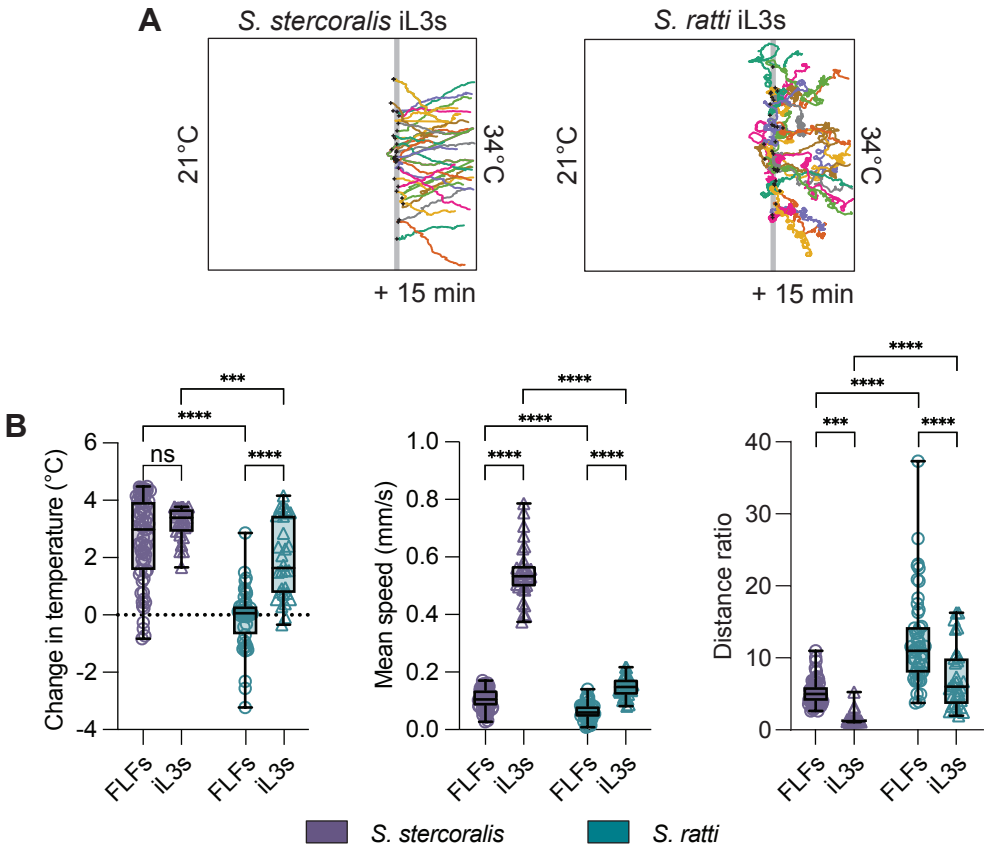

bioRxiv preprint doi: https://doi.org/10.1101/2024.09.12.612595; this version posted September 15, 2024. The copyright holder for this preprint (which was not certified by peer review) is the author/funder, who has granted bioRxiv a license to display the preprint in perpetuity. It is made available under aCC-BY 4.0 International license.

Figure S5

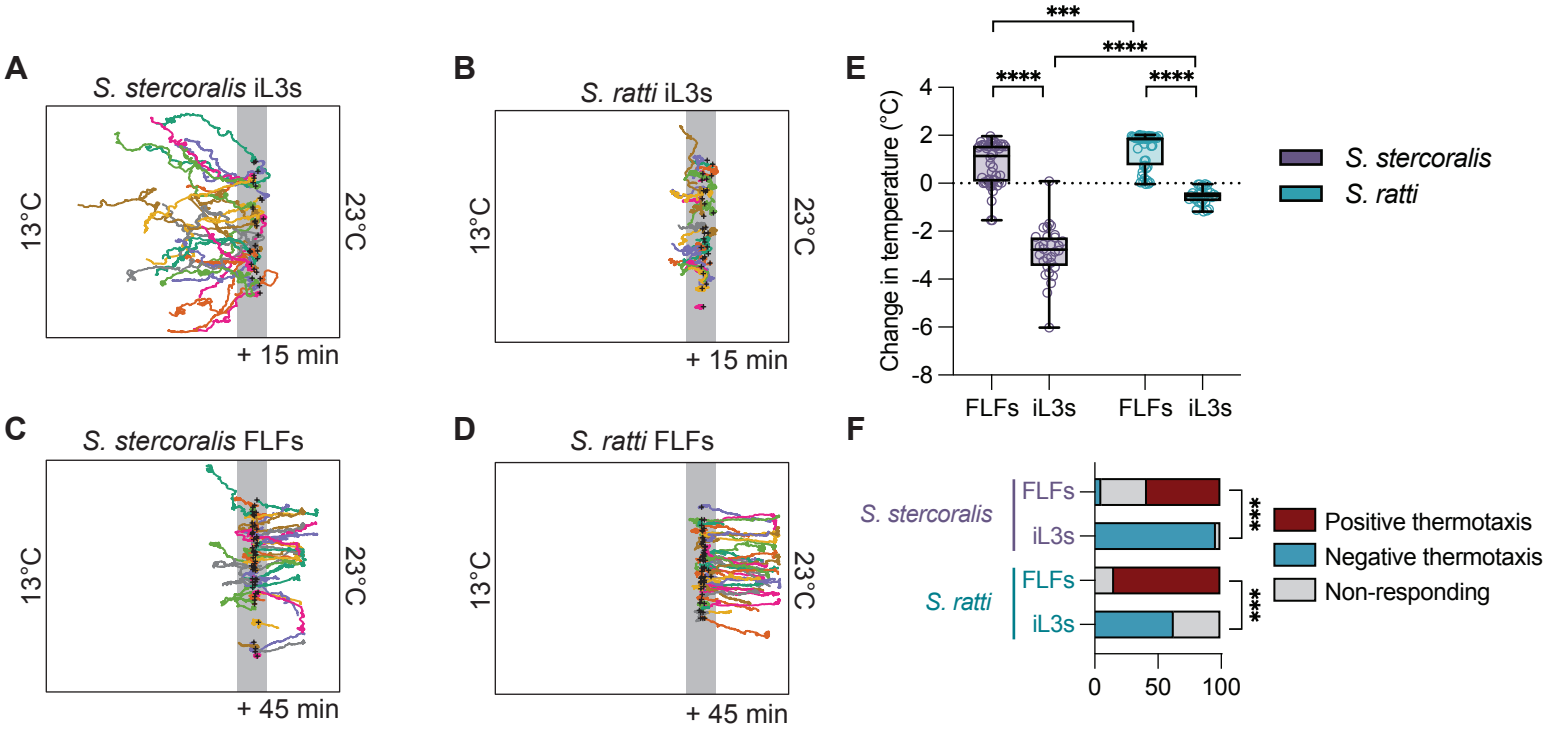

bioRxiv preprint doi: https://doi.org/10.1101/2024.09.12.612595; this version posted September 15, 2024. The copyright holder for this preprint (which was not certified by peer review) is the author/funder, who has granted bioRxiv a license to display the preprint in perpetuity. It is made available under aCC-BY 4.0 International license.

Figure S6

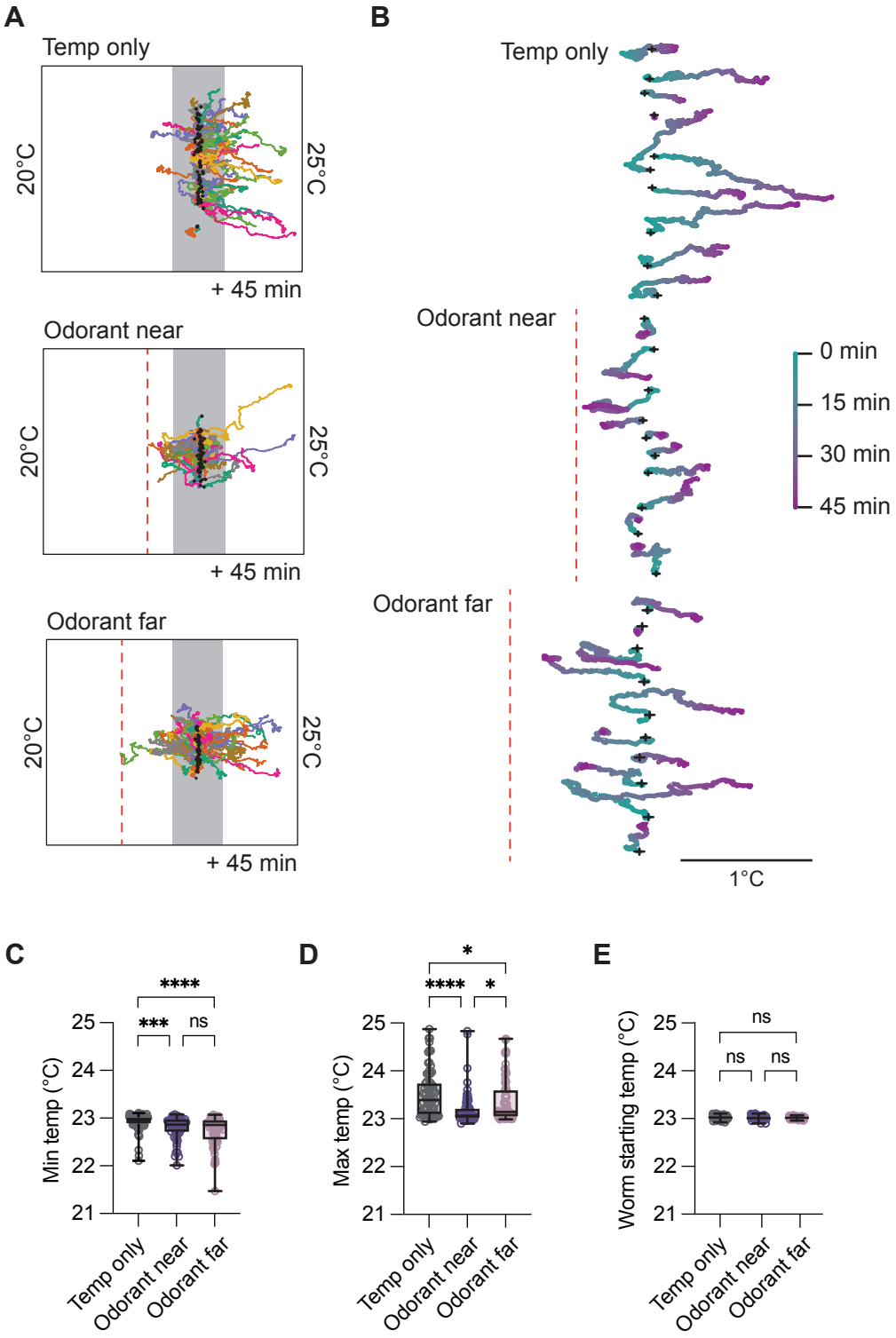

bioRxiv preprint doi: https://doi.org/10.1101/2024.09.12.612595; this version posted September 15, 2024. The copyright holder for this preprint (which was not certified by peer review) is the author/funder, who has granted bioRxiv a license to display the preprint in perpetuity. It is made available under aCC-BY 4.0 International license.

Figure S7

Caenorhabditis elegans adult hermaphrodites

Strongyloides stercoralis free-living females

Strongyloides ratti free-living females

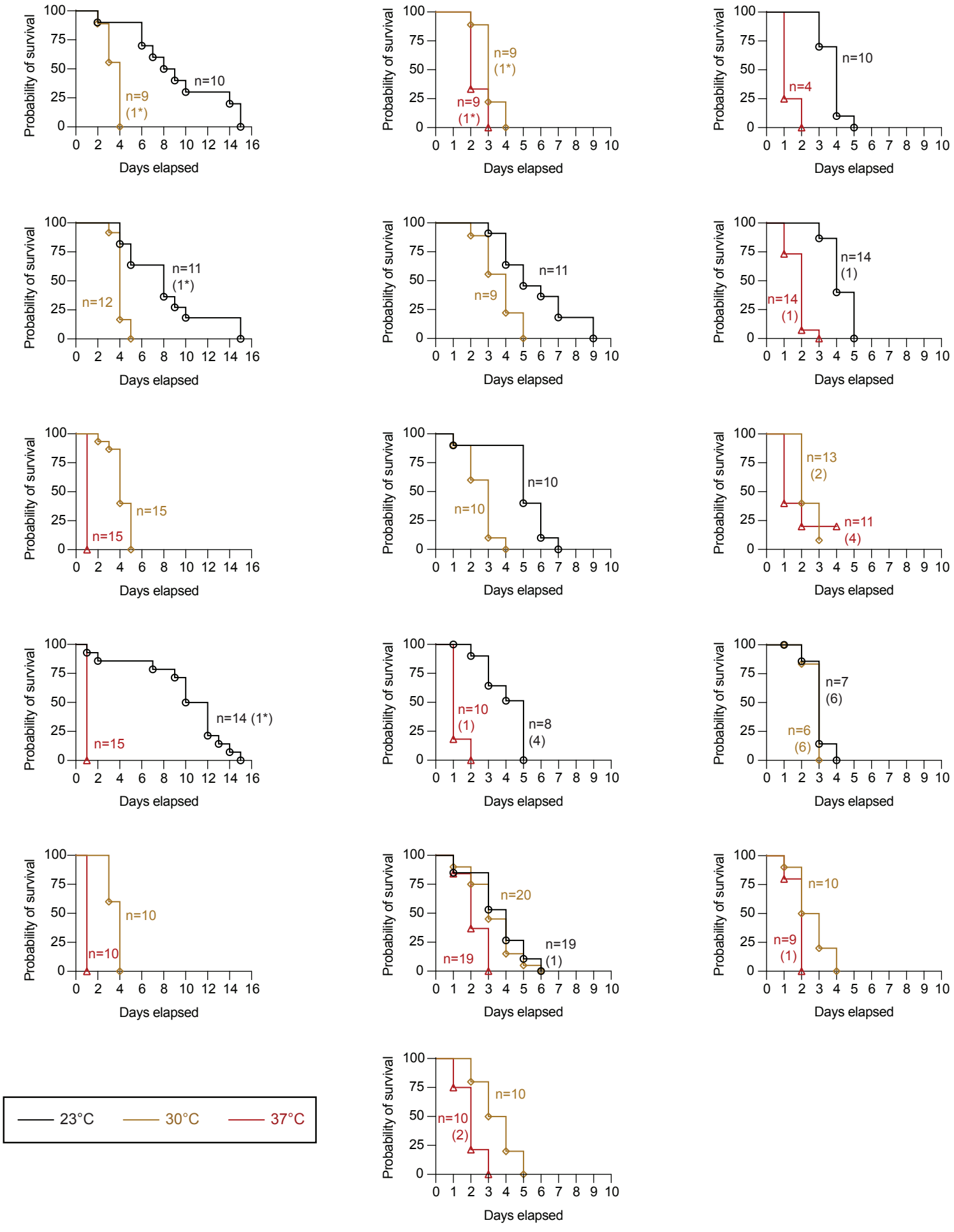

bioRxiv preprint doi: https://doi.org/10.1101/2024.09.12.612595; this version posted September 15, 2024. The copyright holder for this preprint (which was not certified by peer review) is the author/funder, who has granted bioRxiv a license to display the preprint in perpetuity. It is made available under aCC-BY 4.0 International license.

Figure S8

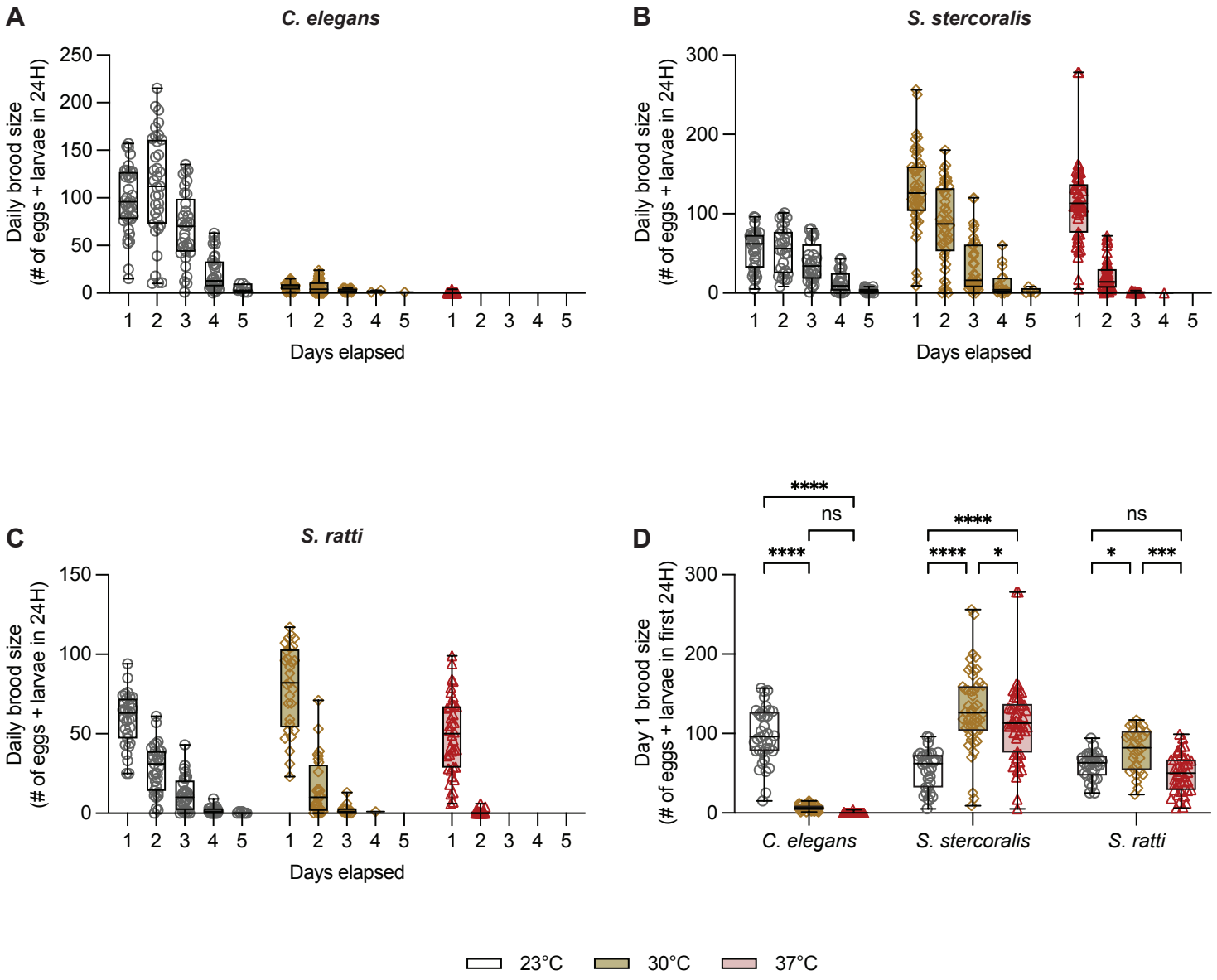

Supplement: 1 [file NIHPP2024.09.12.612595v2-supplement-1.pdf]
